# Chloroform and Ether in Their Medico-Legal Relations

**Published:** 1877-09

**Authors:** A. M. Denig


### ARTICLE VI.

### *Chloroform and Ether in their Medico-Legal Relations.*

Dr. A. M. Denig, (*Ohio Med. Recorder*, Jan., 1877) in an interesting paper before the Columbus Pathological Society, presents the consideration of chloroform and ether in their medico-legal relations, under the following three heads:

1. Can they be administered successfully to persons during natural sleep without awakening them?

2. Can they be forcibly administered for criminal purposes, in opposition to the will of the person to whom they are given?

3. Can a person give competent testimony as to what occurred during the anæsthetic state?

For convenience sake, he discusses them in reverse order, and arrives at the following conclusions:

1. In the third question, the person is supposed to take the anæsthetic voluntarily. Now the action of chloroform is divided into three stages—first, that in which the symptoms resemble alcoholic intoxication, in which the patient is often noisy and talkative and gives play to the emotions, etc. This stage is usually very short and rapidly passes into the second, in which the consciousness is wholly lost, and in which the patient lies passive and helpless. In the third case narcosis is profound, the breathing stertorous, muscular relaxation extreme, and complete abrogation of all reflex actions. Every one who is at all acquainted with the phenomena excited by chloroform and ether knows that there is a time during the first stage of the intoxication in which pain is nearly or quite abolished, and yet the consciousness of the patient is almost intact. At no time during this stage, however, is there a complete abolition of the power of volition; the patient is not deprived of the power of resistance, and they will frequently tell you that they were entirely conscious of all that transpired, and yet felt little or no pain if any operative procedures had then been made. Therefore there was a voluntary act of the will in allowing the operation to proceed, with a perfect consciousness of the fact that they could have prevented it had they so desired. Now if, during this time, any criminal act takes place, a passive, unresisting submission would imply her entire acquiescence in the act, and make her "*participio criminis*." But, when the power of resistance or outcry is abolished, the consciousness of a real motive for it is lost also. If, now, an outrage be perpetrated or attempted on a woman, she cannot be aware at the time of the liberty which is being taken with her person, and her statements should be entirely repudiated as either the product of her imagination or a deliberate and wicked misrepresentation for sinister and selfish purposes.

2. Can chloroform be administered forcibly? Ordinary experience in the administration of anæsthetics teaches us that none of those articles can be forcibly administered with felonious intent without the exercise of a force sufficient to accomplish the robbery or other contemplated crime without resorting to their use, and that, when such an allegation has been made, a careful examination of the facts will unequivocally prove a collusion between the parties—the assailants and the assailed.

3. Can chloroform be administered during sleep? Besides a number of successful cases scattered through the medical journals, the first systematic experiments made to answer this question are those of M. Dolbean, surgeon of Beaujon Hospital, Paris. Three different series of attempts were made by him, assisted by his "internes," and from his account it is obvious that the more the experimenters became familiar with the "*modus faciendi*" the number of persons successfully anæsthetized proportionately augmented; at first it was one in four, then two in six, then six in nine.

The experiments were made publicly, in the surgical wards, upon patients of all ages, and twelve M. of chloroform were given at a time upon a napkin, held some distance from the person's face. As out of the twenty-nine subjects on which these experiments were tried ten—more than one-third—were successfully anæsthetized, we may say that it is difficult, but it is certainly possible, to render a person insensible by the use of chloroform vapor administered during sleep. Accordingly, the expert must, in justice, declare that it is possible, if it is not easy, to render a person insensible by chloroform during a natural sleep, in order that he may be made the victim of a criminal assault.—*Detroit Med. Journal.*

